# The stability of care preferences following acute illness: a mixed methods prospective cohort study of frail older people

**DOI:** 10.1186/s12877-020-01725-2

**Published:** 2020-09-29

**Authors:** S. N. Etkind, N. Lovell, A. E. Bone, P. Guo, C. Nicholson, F. E. M. Murtagh, I. J. Higginson

**Affiliations:** 1 Cicely Saunders Institute, Florence Nightingale Faculty of Nursing Midwifery and Palliative Care, King’s College London, Bessemer Road, London, SE59PJ UK; 2 Department of Public Health and Primary Care, University of Cambridge, Cambridge, UK; 3 School of Nursing, Institute of Clinical Sciences, College of Medical and Dental Sciences, University of Birmingham, Birmingham, UK; 4 St Christopher’s Hospice, London, UK; 5 University of Surrey, Faculty of Health and Medical Sciences, Guildford, UK; 6 Wolfson Palliative Care Research Centre, Hull York Medical School, University of Hull, Hull, UK; 7 King’s College Hospitals NHS Foundation Trust, London, UK

**Keywords:** Patient preference, Frail elderly, Aged, Patient-centered care, Palliative care, Cohort studies

## Abstract

**Background:**

Patient preferences are integral to person-centred care, but preference stability is poorly understood in older people, who may experience fluctuant illness trajectories with episodes of acute illness. We aimed to describe, and explore influences on the stability of care preferences in frail older people following recent acute illness.

**Methods:**

Mixed-methods prospective cohort study with dominant qualitative component, parallel data collection and six-month follow up. Study population: age ≥ 65, Rockwood Clinical Frailty score ≥ 5, recent acute illness requiring acute assessment/hospitalisation. Participants rated the importance of six preferences (to extend life, improve quality of life, remain independent, be comfortable, support ‘those close to me’, and stay out of hospital) at baseline, 12 and 24 weeks using a 0–4 scale, and ranked the most important. A maximum-variation sub-sample additionally contributed serial in-depth qualitative interviews. We described preference stability using frequencies and proportions, and undertook thematic analysis to explore influences on preference stability.

**Results:**

90/192 (45%) of potential participants consented. 82/90 (91%) answered the baseline questionnaire; median age 84, 63% female. Seventeen undertook qualitative interviews. Most participants consistently rated five of the six preferences as important (range 68–89%). ‘Extend life’ was rated important by fewer participants (32–43%). Importance ratings were stable in 61–86% of cases. The preference ranked most important was unstable in 82% of participants.

Preference stability was supported by five influences: the presence of family support; both positive or negative care experiences; preferences being concordant with underlying values; where there was slowness of recovery from illness; and when preferences linked to long term goals. Preference change was related to changes in health awareness, or life events; if preferences were specific to a particular context, or multiple concurrent preferences existed, these were also more liable to change.

**Conclusions:**

Preferences were largely stable following acute illness. Stability was reinforced by care experiences and the presence of family support. Where preferences were unstable, this usually related to changing health awareness. Consideration of these influences during preference elicitation or advance care planning will support delivery of responsive care to meet preferences. Obtaining longer-term data across diverse ethnic groups is needed in future research.

## Background

Care preferences, defined as ‘what people want from their care’, [1] may relate to the purpose of care, care context, involvement in care, or care relationships [2, 3]. A core tenet of person-centred care is that it takes into account and is responsive to patient preferences [2, 4].

However, care preferences are not always stable [5–7]. This may particularly be the case in older populations as chronic conditions, multimorbidity and frailty increase, because preferences may be more liable to change during the course of a long and unpredictable illness trajectory [8–10]. It is therefore important to understand preference stability in older people.

Frailty may affect preference stability [11], since it is associated with fluctuating function and frequent acute illness episodes [12–15]. The health status changes and care experiences associated with acute illness may destabilise preferences [7, 16, 17].

Longitudinal studies of preferences have consistently found that whilst most preferences are stable, some people do change their preferences over time [5, 18, 19]. Preferences among older people are influenced by individual, illness, and contextual factors, particularly family support [7]. These factors may affect preference stability. In the context of recent acute illness, frail older people may focus their care preferences on the purpose of care - what they wish their care to achieve [16], but few studies have examined why preferences do or don’t change over time [7].

Clinically, knowledge of preference stability patterns and their influences would enable timely reassessment of preferences. It would facilitate advance care planning (ACP), which seeks to identify people’s preferences for future care [20]. ACP is increasingly recognised as an iterative process [20], with complex models of planning more likely to result in care meeting preferences at the end of life [21], but it nevertheless assumes a degree of preference stability. Further evidence is needed to underpin this assumption and optimise ACP models.

To deliver responsive care, we need to understand preference stability in the frail older population, and what influences the stability of preferences following acute illness. In this study, we aimed to describe, and explore influences on, the stability of and changes in preferences for purpose of care in frail older people with recent acute illness.

## Methods

### Study design and theoretical framework

We undertook a mixed-methods prospective cohort study (the IARE II study) with a convergent design and dominant qualitative component [22, 23]. This incorporated questionnaires to describe preference stability/change, conducted in parallel to serial interviews exploring influences on preference stability [22]. Existing theoretical models of care preferences in frail older people [16], and response shift [17], were used as frameworks to explore influences on preference stability. Ethical approval was received from the UK Health Research Authority (reference 16/LO/2048).

### Setting

Participants were recruited from two acute hospitals, one sub-acute hospital, and one acute community service in South London (UK) between February 2017 and July 2018. One acute hospital and the acute community service were in city centre locations with diverse and relatively socioeconomically deprived populations. The other hospitals were in suburban areas with less deprived populations [24].

### Sampling and recruitment

The inclusion criteria were: Age ≥ 65; Rockwood Clinical Frailty Score (CFS) ≥5 [25]; and acute illness requiring a) hospital admission or b) two acute care attendances in the last 6 months. Those receiving specialist palliative care, and those lacking capacity with no personal consultee (a friend or relative willing to provide written approval on the participants behalf should they lack capacity to consent) were excluded. Sample size was based on ability to accurately estimate the prevalence of each preference, and 20% attrition due to death and illness was assumed in line with previous longitudinal research in similar populations [26]. Potentially eligible participants were identified by clinicians at each site, who gained consent to contact. Following confirmation of eligibility, researchers approached the participant, explained the study, and allowed 24 h consideration before returning to take written or witnessed verbal consent. A purposive maximum-variation sub-sample of participants (sampling criteria: age, Australian modified Karnofsky Performance Status (AKPS), number of hospitalisations, and living alone vs living with someone) undertook qualitative interviews.

### Data collection

Participants answered three face-to-face questionnaires at 12-week intervals over a six-month follow up period. The questionnaires asked about six preferences regarding the purpose of care, henceforth referred to as ‘care preferences’. These were chosen based on literature review [3], with input from a patient and public involvement (PPI) project advisory group with whom we discussed possible categories of care preference. The preferences were: to extend life; to improve quality of life for the time they had left; to remain as independent as possible; to be comfortable; to support those close to them; and to stay out of hospital. Participants could specify one additional preference of their own choosing (supplementary information 1). We were interested in two forms of preference - rating and ranking. Firstly, participants rated the importance of each of the above preferences on a 0–4 Likert scale; 0 marked as ‘unimportant’, 4 as ‘extremely important’. Secondly, they ranked the single most important preference. Measures of health problems and concerns, function, and service use were included in the questionnaire (supplementary information 1.) Elixhauser comorbidity score and health service use were collected from hospital notes [27], and researchers Identified frailty and overall functional status using the CFS [25], and AKPS [28].

The qualitative sub-sample additionally contributed three serial in-depth qualitative interviews to explore influences on their preferences. The interviews were conducted by one male researcher in a place of the participants choosing; follow up interviews were conducted by the same researcher as the baseline interviews. The topic guide covered experiences of illness and care, preferences and influences on preferences, and concerns about the future. Follow up interviews used the same topic guide, additionally focusing on changes from baseline experiences. Further details of data collection for the qualitative interviews, including the full topic guides, are reported elsewhere [16].

### Analysis

#### Data management

Questionnaires were entered into an SPSS database (IBM SPSS Statistics for Windows, Version 22.0. Armonk, NY: IBM Corp.) and checked with 10% double-data entry. Missing preferences were not imputed. If two researchers (SNE & AB) agreed that a preference of participant’s own choosing corresponded to one of the six pre-specified preferences, this was re-categorised accordingly. Qualitative interviews were recorded and transcribed verbatim, then uploaded to NVIVO software (QSR International Pty Ltd. Version 11, 2015) for analysis [16].

#### Quantitative analysis

Our analysis was descriptive. At each time point we described distributions of the importance rating of each preference and the preference ranked most important using frequencies and proportions. Preferences were considered to be rated ‘important’ if a response of ≥3 out of 4 was given on the Likert scale. Then, preference stability was described as follows:

i) The importance rating of each preference was considered stable when there was ≤1 point change in importance across all study time points, in line with previous methodology [29]. We calculated the number and percentage of participants with stable vs. changing preferences, and the number/percentage whose preferences increased or decreased in importance during the study.
ii) The preference ranked as most important was considered stable if the same preference was ranked as most important at every available measurement.

#### Qualitative analysis

Serial qualitative interviews were analysed thematically [30]. To maintain participant narratives, transcripts were initially read in chronological order and key information and reflective notes were summarised in a case document for each participant [31]. Transcripts were coded by one researcher (SNE); three were coded independently by another researcher (NL). The coding domains were based on our previously reported model of preferences, which was formulated from the baseline interviews of the participants in this study [16], and inductive coding of serial interview transcripts was underpinned by these domains, extending the baseline model to incorporate influences on preference stability.

#### Integration

Influences on the preference stability patterns identified in the questionnaire data were sought in the coded qualitative data. This stage incorporated both qualitative and quantitative data, in an explanatory integration analysis [22], corresponding to ‘following a thread’ between datasets [32]. The responses of participants who provided both qualitative and quantitative data were compared in a mixed-methods matrix to explore preference stability patterns at an individual level [32]. To enable integration, the questionnaire preferences expressed by qualitative participants during the study were classified overall as *stable* (the preference ranked most important was the same at all measurements, and the importance rating of the preference ranked most important was stable; *semi stable* (The preference ranked most important was stable, but the importance rating of the preference ranked most important changed (or vice versa); or *Unstable* (Both the preference ranked as most important, and the importance rating of preferences ranked most important changed during the study). See supplementary information 2 for full details.

## Results

### Participants

**Fig. 1:**
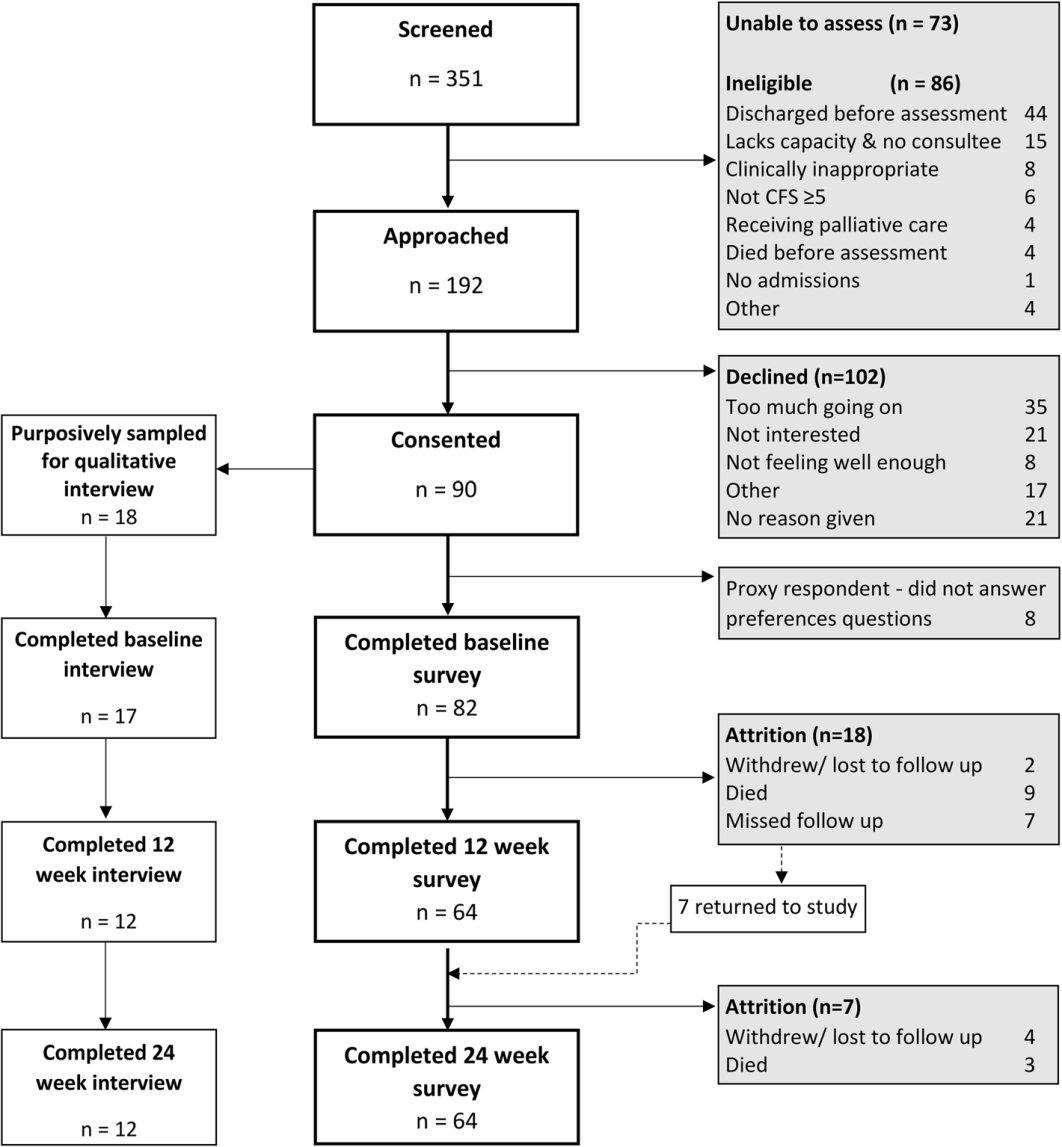
progress through study in line with STROBE reporting guidance [33].

45% of 192 eligible patients consented to participate; 82/90 participants completed the baseline preferences questionnaire. Twelve participants died during the study, and 7 participants withdrew or were lost to follow up, meaning that 64 participants (78%) completed the study (Fig. 1). Participant characteristics are detailed in Table 1.

**Table 1:** Characteristics of study participants

Characteristic	All participants (*n* = 82)^1^	Participants who contributed qualitative interviews^2^ (*n* = 17)
Age (median (Interquartile range (IQR)))	84 (79–89)	82 (81–86)
Gender		
Male n (%)	30 (37)	8 (47)
Female n (%)	52 (63)	9 (53)
Number of hospital admissions (median (IQR))		
In 6 months prior to study	2 (1–3)	1 (1–3)
During study	1 (0–2)	1 (0–2)
Elixhauser comorbidity score (median (IQR))	3 (2–5)	4 (3–5)
Presence of cognitive impairment^3^ n (%)	19 (23)	4 (22)
CFS^4^ (median (IQR))		
Baseline	6 (5–6)	6 (5–7)
12 weeks	6 (5–6)	6 (5–6)
24 weeks	6 (5–6)	6 (5–7)
AKPS^5^(median (IQR))		
Baseline	50 (50–60)	50 (50–60)
12 weeks	60 (50–60)	60 (50–60)
24 weeks	60 (50–60)	50 (40–60)
Income status n (%)		
Living comfortably on current income	41 (50)	9 (53)
Coping on current income	32 (39)	8 (47)
Difficult on current income	4 (5)	0 (0)
Very difficult on current income	1 (1)	0 (0)
Don’t know	2 (2)	0 (0)
Prefer not to say	2 (2)	0 (0)
Religious n (%)		
Yes	60 (73)	10 (59)
No	21 (26)	7 (419)
Missing	1 (1)	0 (0)
Living status n (%)		
Lives alone	43 (52.4)	8 (47)
Lives with someone	39 (47.6)	9 (53)
Ethnicity n (%)		
White British	70 (85)	17 (100)
White other	2 (2)	0 (0)
Irish	3 (4)	0 (0)
Caribbean	4 (5)	0 (0)
African	1 (1)	0 (0)
Other	1 (1)	0 (0)
Missing	1 (1)	0 (0)

^1^90 participants consented, but 8 required a proxy respondent and could not answer the preferences questions. Details of the 82 participants who answered the baseline survey are reported here. ^2^One participant was sampled for qualitative interviews but was unable to complete. ^3^Includes dementia, delirium, and cognitive impairment without formal diagnosis. ^4^CFS = Rockwood Clinical Frailty Scale ^5^AKPS = Australian modified Karnofsky Performance Status

### Patterns of preferences and their stability

#### Importance rating

Most participants consistently rated five of the six preferences as important (68–89%) throughout the study (Table 2). The exception was preference A ‘to extend life’ which was rated important by fewer participants (32–43%), following a U-shaped distribution. See supplementary information 3 for additional detail of the rating distributions. The importance of ‘to extend life’ was stable in only 61% of participants, whereas the other five preferences were stable in ≥80% of participants. D ‘to be comfortable’ was most frequently stable (86%). The proportion of ‘don’t know’ answers was 3%; more participants (8%) reported ‘don’t know’ answers for ‘extend life’ than other preferences. Four preferred not to answer for each preference, and on average 5% (range 3–10%) of data were missing (supplementary information 4)**.**

**Table 2:** Importance rating of preferences

		A. Extend life	B. Improve quality of life	C. Remain independent	D. Be comfortable	E. Support those close to me	F. Stay out of hospital
**Percentage rating each preference as important**^**a**^							
Baseline (*n* = 82)	%	43	81	86	89	77	82
12 weeks (*n* = 64)	%	32	75	82	89	75	82
24 weeks (n = 64)	%	39	76	79	82	68	78
**Stability of importance ratings**^b^							
Stable importance rating at all measurements	n (%)	33/54 (61)	47/59 (80)	54/63 (86)	54/63 (86)	51/62 (82)	51/63 (81)
Unstable importance rating: importance increased	n (%)^c^	14 (23)	7 (12)	4 (6)	5 (8)	4 (6)	6 (10)
Unstable importance rating: Importance reduced	n (%)^c^	13 (21)	7 (12)	5 (8)	5 (8)	9 (1)	6 (10)

^a^Importance was rated on a 0–4 Likert scale. ‘Important’ defined as a score of ≥3. Don’t know/prefer not to say answers were included, missing answers were excluded.

^b^Stability = change of ≤1 point in importance rating of a preference across all data points. Denominator = all participants who provided ≥2 measurements for each preference.

^c^Some participants reported both an increase of > 1 point and a decrease of > 1 in preference importance rating over the three questionnaires. Both have been counted here so percentages add up to more than 100 in some columns

#### Ranking of most important

All preferences were ranked most important by some participants (Table 3). At baseline, preference F ‘stay out of hospital’ was ranked most important by the highest proportion (20%), and D ‘be comfortable’ was ranked most important by the lowest proportion (4%). 11% of participants did not know what was most important, and 4% preferred not to say. The preference ranked most important changed at 66% of opportunities (64% between baseline and 12 weeks, 67% between 12 and 24 weeks), and 82% of participants with ≥2 time-points had at least one change in their most important preference.

**Table 3:** Preference ranked most important during study

Time point	A. extend life	B. Improve quality of life	C. Remain independent	D. Be comfortable	E. Support those close to me	F. Stay out of hospital	G. Other (specify)	Don’t Know	Prefer not to say	missing
Baseline n (%) (*n* = 82)	7 (9)	12 (15)	8 (10)	3 (4)	13 (16)	16 (20)	6 (7)	9 (11)	3 (6)	5 (6)
12 weeks n (%) (*n* = 64)	5 (8)	6 (9)	9 (14)	7 (11)	8 (13)	13 (20)	5 (8)	5 (8)	3 (5)	3 (5)
24 Weeks n (%) (*n* = 64)	6 (9)	7 (11)	8 (13)	7 (11)	8 (13)	9 (14)	8 (13)	5 (8)	4 (6)	2 (3)
Percentage stable^a^ (*n* = 57)	17	11	0	0	25	19	0	–	–	–

^a^Percentage of participants who rated the same preference most important at baseline and at all available follow ups (those with only 1 data point were excluded)

### Influences on preference stability

**Fig. 2:**
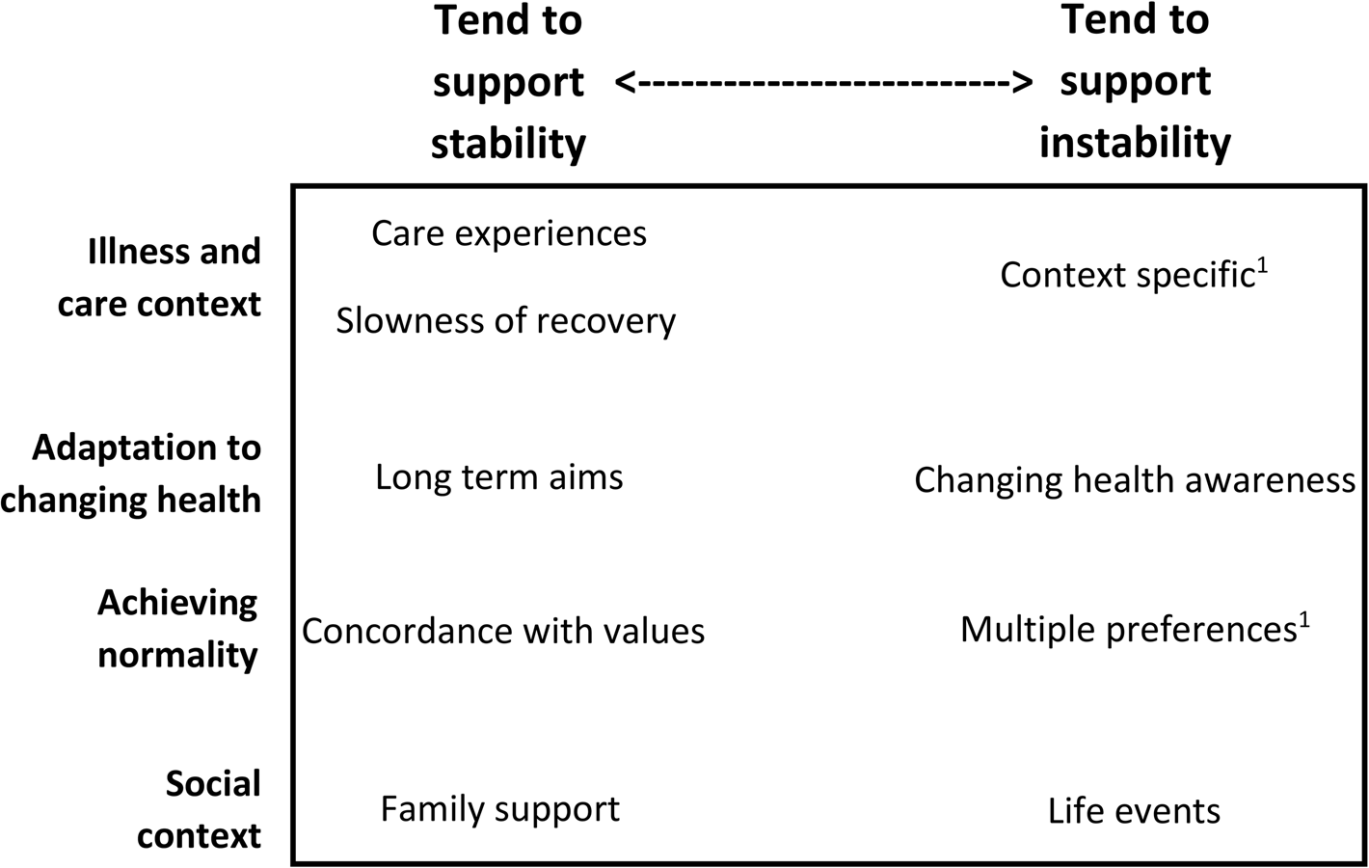
Influences on care preference stability, arranged according to whether they tend to stabilise or destabilise preferences. Key: Position in the figure denotes whether an influence tends to support preference stability (left) or preference instability (right). The influences are categorised according to our model of influences on care preferences in frail older people following acute illness. [18] 1. ‘multiple preferences’ and ‘context specific preferences’ only influenced stability of the preference ranked most important. All other influences affected both the importance rating of preferences, and ranking of the most important

From the serial qualitative interviews, we identified five influences that tended to support the stability of both preference importance ratings, and ranking of the most important preference: good or bad *care experiences*; *concordance with values;* presence of *family support; slowness of recovery;* and *long term goals.* Changes in preferences usually related to one influence: *changing health awareness*, but when they occurred, *life events* also tended to support preference instability*. Multiple preferences,* and *context specific* preferences supported preference instability, but only for the preference ranked most important (Fig. 2). These influences are discussed in detail below. Further exploration of preference stability in the qualitative sub-sample is presented in supplementary information 2.

Notably, participants often expressed preferences inconsistently, for example expressing unstable preferences in their questionnaire responses, but expecting preferences to remain stable in the qualitative interviews. Others thought their preferences would change, but had stable preferences in the questionnaire. This divergence between qualitative and quantitative data suggests a difficulty in considering or articulating preferences, and that preferences were influenced by more than rational conscious choice alone.

#### Influences that support preference stability

##### Care experiences

appeared to affect preferences in all cases. Experiences tended to support preference stability, particularly related to place of care, suggesting that preferences are reinforced by exposure to, and knowledge of the care they relate to. This applied in both directions: where care experiences were good, this tended to confirm participants’ views about the care they were receiving. One participant felt the care they were getting at home was ideal, therefore preferred to remain cared for in this way at home and to avoid hospital. Staying out of hospital was rated highly important in each questionnaire response for this participant.

‘No…No…. if I could be to the end of my days, if I could be looked after the way I am now, both in family and medically, I would say that, I would say I would be happy’

P14, interview 3. Male aged 90 - 94 with stable preferences for staying out of hospital, overall preferences semi-stable

Conversely, several participants reported poor care experiences in hospital which also stabilised preferences in that participants wished to avoid repeated occurrences and therefore to stay out of hospital.

‘Oh, I didn’t like [being back in hospital] at all because from being in there before you know I remember thinking ‘o I hope I haven’t gotta come back here’. Um, so no it wasn’t … I suppose it isn’t a good experience going to hospital is it?’

P9, interview 2. Female aged 85 - 89 with stable preferences for staying out of hospital, overall preferences stable.

##### Concordance with values:

Where preferences aligned with values, they seemed less likely to change, even when participants did not clearly articulate why they held particular values. This frequently related to a desire to maintain independence, or remain at home:

‘I think when you go into hospital you lose a lot of your dignity, your self-reliance… (2 second pause (2s))…you become dependent on other people…. You’d like to be independent, and by losing that, you lose something from life…9s…’

P3, interview 3. Male aged 85 - 89 with stable preferences for remaining independent and staying out of hospital, overall preferences semi-stable.

##### Family support

Having support from family was important to most participants, and participants expressed how support from their family enabled them to live in line with their preferences. One participant expressed this by considering the reverse, stating that if he didn’t have family support, his care preferences would change.

‘Researcher (R): And how would that [not having family around] change things for you?

Participant (P): …Don’t know; be difficult to know really. But um [I] think you’re a bit more inclined to go into a [nursing] home … if you didn’t have a family around you, because at least there you get support’

P12, interview 2. Male aged 80 - 84 with stable preferences for having family support and for place of care, overall preferences semi-stable.

For some, concerns about family also exerted a stabilising influence on preferences. One participant felt that ongoing family concerns were a reason for his stable preference to stay out of hospital:

‘R: you mentioned that staying out of hospital is very important to you, and would remain important to you if you were less well. Can I ask why that is?

P: I think it disrupts the whole family. Not just me, but the whole family and I mean going into hospital as such isn’t an issue as such I’m not afraid of hospitals or anything like that, but I think it disrupts the families quite a lot. It causes people a lot of worry and it isn’t always necessary’

P3, interview 3. Male aged 85 - 89 with stable preferences for supporting those close to him, and for staying out of hospital, overall preferences semi-stable.

##### Slowness of recovery

Slowness of recovery or recurrent illness sometimes facilitated preference stability, as participants existed in a semi-permanent recovery process. Participants largely wanted to ‘get back to normal’ and their preferences regarding independence were influenced by this desire. If they were not making progress towards normality, their preference to recover and achieve normality would tend to remain the same, with a focus on what was immediately important.

‘Yes … I really didn’t expect … pneumonia to have knocked me back for quite so long a period of time … (3s) … because I was sure I’d get back to normal but I’m not.’

P11, interview 3. Male aged 85 - 89 with stable preferences for remaining independent and improving quality of life, overall preferences stable.

##### Long term aims

Some participants’ preferences related to a long term aim, frequently to regain independence. This tended to remain important until either that aim was achieved, another goal superseded it, or the participant became aware that the aim was unachievable.

‘Well, I want to improve so I can care for meself. That’s what I wanna do. But whether that’ll ever be possible I don’t know. I don’t know how you can improve, how you can improve things like that.’

P4, interview 3. Male aged 80 - 84 with stable preferences for remaining independent, overall preferences semi-stable.

Paradoxically, some who expressed long term aims had unstable preferences, possibly because they were less aware of likely changes in their health (see also ‘*changing health awareness’ below)*.

‘… no, no I mean my priority is now as I’ve said before is able to walk better and go out and do things, and um I don’t think that will ever change that’s my priority but it might take a long long time to achieve. A long time.’

P2, interview 3. Female aged 85 - 89. Stable preferences for remaining independent, overall preferences unstable

#### Influences that support preference instability

##### Changing health awareness

Representing development of new understanding about participants’ health state, supported preference instability. A change in health awareness could result in reprioritisation, and therefore change in either preference ranking or rating.

‘….(4s)….Well I suppose they [priorities] have [changed] in a way, because I’m in a **different position**….(3s)… so the priorities are basically to get home and move around the house’

P12, interview 1. Male aged 80 - 84 with unstable preferences for improving quality of life, stable preferences for remaining independent and overall preferences semi-stable

Whilst care experiences by themselves tended to stabilise preferences, sometimes they precipitated changes in health awareness. When this was the case, preferences were more liable to change. This participant had strongly wished to remain at home, but her place of care preference changed to care home:

P: ‘[my preference is]… to be outside [hospital], and… to live a normal life…. But I don’t stand a chance any more.

R: why do you say that?

P: Well there’s such a good pattern of how things have gone. There’s no denying that I’m spending far too much time in hospital’

P13, interview 2. Female aged 70 - 74 with unstable preferences for staying out of hospital, overall preferences semi-stable.

Changing awareness was usually gradual, but occasionally participants experienced larger, more abrupt changes, which could change the importance rating of preferences. One participant realised during the study that independence was no longer possible, and that she needed external support. In her questionnaire data, remaining independent was subsequently rated less important (rating change from 4 to 2 – see supplementary information 2).

‘R: you mentioned that um being, being independent isn’t quite so important to you now… I just wondered why you feel that way?

P: well, I know that I can’t cope on my own really but … (2s) … so … (3s) ... you know’

P5, interview 3. Female aged 80 - 84 with unstable preferences for remaining independent, overall preferences semi-stable.

Preference changes were more closely related to *changes* in awareness than to the level of awareness itself. Participants with high baseline awareness, who had already considered future preferences should their health deteriorate, tended to have stable preferences. One participant had stable preferences, but foresaw future change.

‘I mean I do often think how much longer can I do this, am I going to end up in a home because … you know when you sort of can’t get from there to there or there to there and you think ‘oh how long can I cope doing this’

P9, interview 2. Female aged 85 - 89 with stable preferences for staying out of hospital, overall preferences stable.

Health awareness was particularly relevant in relation to preferences for ‘extending life’. Participants often recognised they had led a full life and felt ambiguous about wanting to live longer. The importance of ‘extending life’ was therefore variable, and depended on participants’ views of their current health status. One participant was aware of a life limiting illness, and accepted her short prognosis, though her preferences for extending life continued to fluctuate.

‘… It [dying] doesn’t worry me, doesn’t worry me because I know it’s going to happen eventually. I mean everyone’s gonna go eventually aren’t they? So … (1.5s) … I’m no spring chicken... I’ve had a good innings’

P5, interview 1. Female aged 80 - 84 with unstable preferences for extending life, overall preferences semi-stable.

##### Life events

Sometimes changed the importance rating of preferences. One participant considered it extremely important to support those close to him. However following the death of his wife, the importance of this aspect reduced.

Interview 2. ‘Well she’s got dementia and that’s taking its course, well that’s there. … and um she um … (3s) it shows. I try and visit her … once a week. We have lunch in the [care] home together, I have whatever everybody else is having. … Um ... and ... as I say I miss her quite a lot’

Interview 3. ‘… Since you came last I think my wife was … in a care home um sadly she died…’

P8, interviews 2 and 3. Male aged 90 - 94, with unstable preferences for supporting those close to him, overall preferences unstable.

##### Context specific preferences

Preferences might be linked to a particular situation, experience, or point in time; when the situation changed, so did the preference. This applied only to the preference which was ranked most important, and mainly related to hospitalisation. One participant had a stable preference to avoid hospitalisation during the study, but she described how this had previously been overridden by pain:

‘I don’t want to go to in hospital, but I was in so much pain, I think in the end I thought to myself ‘Do what you like’

P17 interview 1. Female aged 75 - 79 with unstable preferences for being comfortable, overall preferences stable

Another participant recognised that whilst they didn’t want to go back to hospital, in different circumstances they might have to go:

‘Oh well quite honestly I wouldn’t I wouldn’t want to go back to hospital again. But of course if I had to, well that’s different’

P14, interview 3. Male aged 90 - 94 with stable preferences for staying out of hospital, overall preferences semi-stable.

Some preferences were specific to a point in time. Participants who were reluctant to consider future preferences, focusing rather on the day to day, tended to have less stable preferences. If future preferences had not been considered, a new experience or health change might result in a change in awareness and subsequent re-evaluation of preferences.

‘No, you can’t think about getting worse, otherwise you’ll end up doing, getting worse, you know?’

P2, interview 3. Female aged 85 - 89 considering future care preferences, overall preferences unstable.

Conversely, those who thought extensively about the future were less likely to encounter an unexpected situation and their preferences were usually more stable.

‘I can’t see it [health] getting better cos obviously it won’t get better… it won’t get any better so I suppose with me it’s just a case of going on as long as you can like this every day hoping it doesn’t get worse. But one day it will get worse. In what way I don’t know, but it will won’t it, because what else is there.’

P9, interview 3. Female aged 85 - 89 considering future care preference, overall preferences stable.

##### Multiple preferences

As per Table 2, participants usually considered multiple preferences important. It was often a struggle for participants to decide which was the most important, which led to instability of preference ranked most important. One participant considered ‘getting better’, ‘living day to day’, ‘social contact’ and future security re: place of care important.

'You know my one aim is to get better. To start having a little bit of … enjoyment my retirement what’s left I don’t know how much longer is left but not to be a burden on anybody that’s my priority. And of course the second thing has already been dealt with I feel safe in the knowledge I won’t be put out on the streets I won’t be at the mercy of any of these care homes.’

P7, interview 1. Female aged 85 - 89, preference stability not assessed.

## Discussion

For the first time, this study has described and explored influences on the stability of care preferences in a frail older population following acute illness. Five of the six care preferences studied were consistently rated as important over time. The importance of extending life followed a U-shaped distribution, fewer participants rated it as important, and its importance was less stable. Which preference was ranked as most important was unstable for most participants. Positive or negative care experiences and the presence of family support, alongside slowness of recovery, long term aims, and overlap with values, tended to stabilise preferences. Conversely, a change in health awareness tended to destabilise preferences, alongside life events, context specific preferences, and the existence of multiple preferences.

We found that the importance rating of preferences was relatively stable during the study. This is of consequence because it means that even following the upheaval of acute illness, most preferences remained stable. Indeed care experiences themselves tended to reinforce existing preferences, e.g. a bad experience might reinforce a preference to avoid hospital, whilst a good experience might tend to reinforce preferences to receive the same care in future. One clinical implication of this finding is that it supports the value of recording preferences in advance, something which is infrequently done in the older population [34]. However some preferences did change, particularly how important it was for participants to extend their lives, which changed in both directions, meaning that such preferences should be revisited over time including in the period following acute illness. Since the importance rating of preferences was more stable than ranking of the most important, questions that avoid asking about the relative importance of preferences may be more useful in the clinical assessment of preferences unless it is beneficial to detect subtle preference changes. Existing forms of words could be amended, for example Chochinov’s ‘What are the things at this time in your life that are most important to you or that concern you most?’ could be amended to ‘What are the things that are important to you or that concern you about your care?’ [35] Conversely, if it is important to detect even subtle preference changes, use of a relative ranking of preferences ‘what is most important’ may be more useful.

Changes in health awareness, representing participants’ overall understanding of their health status and the severity of their illness tended to support preference change, possibly through a process of reframing expectations as participants realised recovery from acute illness might not return them to a ‘normal’ level of function [17]. Care preferences might therefore change as participants sought a ‘new normal’ [16]. This is consistent with previous research in advanced cancer, where greater awareness of terminal illness was associated with increased preferences for symptom focused over life prolonging care [36]. Considering the level of health awareness may therefore be useful clinically to identify the best time to conduct advance care planning, since those with a high level of health awareness and readiness to discuss the future are more likely to have stable preferences [37], whereas those with lower or changing awareness may have less stable preferences. However assessment of awareness is itself complicated by the fact that multiple awareness contexts may exist within and between individuals at any given time [38]. Evolving awareness might in some cases involve a shift from closed to open awareness, which may not actually involve any new information, but rather an ability or willingness to accept what is already tacitly known but not spoken about [39]. It is therefore important to be vigilant for changes in health awareness at any stage of an illness. In some instances, information provision itself may change health awareness and lead to preference change [40]. Therefore reassessment of preferences following information provision may also be appropriate, though the exact timing of when to reassess preferences following information provision is an area for future research.

We found that the majority of participants expressed stable importance ratings of a preference, whereas the majority had unstable most-important preferences; this may represent different thresholds for change. Response shift theory would deem changes in the importance rating of a preference, and changes in the most important preference to both be types of reprioritization or goal-reordering, which may occur following acute illness events [41]. The importance rating of a preference may overlap with underlying values, and be linked to social support, (antecedents in response shift) which are stabilising influences. Conversely, the most important preference is usually dependent on choosing between multiple preferences, all of which may be rated as important, making it less stable. It is possible therefore that where preferences are concerned, there are two types of reprioritisation response shift – *relative* reprioritisation, where the preference ranked most important changes in relation to other preferences at a low threshold; and *absolute* reprioritisation where the importance rating of a preference changes at a higher threshold. This distinction develops response shift theory, and implies that the importance rating of preferences, and ranking of the most important preference should be considered distinct concepts by researchers and clinicians. Clinicians should also be aware that responses to tools such as the ReSPECT process, which asks patients to rank one preference (length of life) relative to another (quality of life) [42], may need to be regularly reassessed, since relative reprioritisation could easily occur.

### Strengths/weaknesses

The inferences of this study are strengthened by its mixed-methods design with full integration of methods during analysis. Collection of two forms of data from the same individuals enabled a convergent design, and it was possible to look directly for qualitative explanations of quantitative responses. We recruited a balanced sample across multiple sites in urban and suburban contexts with differing levels of social deprivation. This broadens the potential transferability of findings. However the study sample, particularly the qualitative sub-sample, did not achieve the ethnic diversity of the local population. Though based on literature review and appraised by PPI representatives, the preferences questions were not psychometrically validated. This limits the conclusions that can be drawn from the data, as construct validity or test-retest reliability cannot be assumed. The preference categories themselves were broad in nature, and it could be argued that, for example, most people would always consider ‘being comfortable’ to be important. This may have made it more difficult for us to identify preference changes, though some people did change their preferences in all cases. The literature review on which the preferences categories were based was focused on ‘goals’ rather than ‘preferences’ and it is possible that these concepts did not completely align, however when considering the overall purpose of care, preferences and goals would be expected to fall into similar categories.

We used a qualitative dominant mixed-methods paradigm; this, combined with a small number of complete cases, meant that we could not test for associations between preference stability and other variables, as others have done [43]. This limits the inferences that can be drawn. However, we did not consider enough was known about influences on preference stability to reliably identify relevant independent variables. Future research could investigate the association of preference stability with some of the influences identified in our analysis, for example degree of care experience. The study duration meant that there was limited time to identify preference change, however a longer study may have incurred unacceptable attrition in this frail population with poor prognosis.

## Conclusion

The importance rating of most care preferences remains stable following acute illness in the frail older population, but preferences for extending life are less important and less stable over time. The preference ranked most important is unstable. A change in health awareness tended to support preference change, so reassessment of preferences after any change in health awareness may be useful for clinicians when eliciting care preferences or seeking to undertake advance care planning.

## Supplementary information

**Additional file 1. Supplementary information 1: **Preferences questions used, and additional study measures. **Figure S1**. Wording and presentation of preferences questions. **Table S1**. Other measures used in this study. **Supplementary information 2:** Details of qualitative participants and their care preferences. **Table S2**. Details of qualitative participants. **Table S3**. Mixed-methods matrix*.* Combined qualitative and quantitative data. Illustrating influences on preference stability patterns. **Table S4**. Qualitative participants’ care preferences at each time point. **Supplementary information 3:** Distributions of importance ratings of preferences. **Figure S2**. Histograms showing how the importance ratings of each preference were distributed at each time point. **Supplementary information 4:** Missing data report. **Table S5**. Unit non- response (missing questionnaires). **Table S6**. Item non-response for most- important preference. **Table S7**. Item non-response for importance rating of each preference.

## Data Availability

The datasets used and analysed during the current study are available from the corresponding author on reasonable request.

## Abbreviations

ACP: Advance Care Planning
AKPS: Australian Modified Karnofsky Performance Status
CFS: Rockwood Clinical Frailty Scale
IARE II: The International Access, Rights and Empowerment II Study
PPI: Patient and Public Involvement
ReSPECT: Recommended Summary Plan for Emergency Care and Treatment
