# Supplementary material for: The stability of care preferences following acute illness: a mixed methods prospective cohort study of frail older people
Source: BMC Geriatr. 2020 Sep 29;20:370. doi: 10.1186/s12877-020-01725-2 (PMC7523327; doi:10.1186/s12877-020-01725-2)
Supplement: Supplementary file 1 — Additional file 1. Supplementary information 1: Preferences questions used, and additional study measures. Figure S1. Wording and presentation of preferences questions. Table S1. Other measures used in this study. Supplementary information 2: Details of qualitative participants and their care preferences. Table S2. Details of qualitative participants. Table S3. Mixed-methods matrix. Combined qualitative and quantitative data. Illustrating influences on preference stability patterns. Table S4. Qualitative participants’ care preferences at each time point. Supplementary information 3: Distributions of importance ratings of preferences. Figure S2. Histograms showing how the importance ratings of each preference were distributed at each time point. Supplementary information 4: Missing data report. Table S5. Unit non- response (missing questionnaires). Table S6. Item non-response for most- important preference. Table S7. Item non-response for importance rating of each preference. [file 12877_2020_1725_MOESM1_ESM.docx]

**Supplementary information 1.** **Preferences questions used in this study, and additional study measures.**

Supplementary figure 1. Wording and presentation of preferences questions

1. **In situations of serious illness with limited time to live, difficult decisions may need to be made and some things may need to be prioritised over others.** **In this situation, how important would each of the following be to you?**

| Answer options | How important? | | | | |
| --- | --- | --- | --- | --- | --- |
|  | (Un-  important) | | | (Extremely  important) | |
| 1. To extend life | 0 | 1 | 2 | 3 | 4 |
| 1. To improve quality of life for the time you had left | 0 | 1 | 2 | 3 | 4 |
| 1. To remain as independent as possible | 0 | 1 | 2 | 3 | 4 |
| 1. To be comfortable | 0 | 1 | 2 | 3 | 4 |
| 1. To support those close to you | 0 | 1 | 2 | 3 | 4 |
| 1. To stay out of hospital | 0 | 1 | 2 | 3 | 4 |
| 1. Other (please specify)..………………….……… | 0 | 1 | 2 | 3 | 4 |

**2. Of the above answer options,**

**for you, which ONE is the *most* important:** (write the letter A-G)………

**for you, which ONE is the *least* important:** (write the letter A-G) ..…….

| **PATIENT DATA** | |
| --- | --- |
| Category | Measure |
| Selected Demographics | Including questions about ethnicity, living status |
| Symptoms/ Concerns | IPOS – 7 days version^1^. EQ5D^2^ |
| Frailty | FRAIL Scale^3^ |
| Patient Preferences | Preference questions based on systematic review (as above) |
| Patient Experience | Picker Institute patient experience questions^4^ |
| Service use | Client Services Receipt Inventory^5^ |
| **RESEARCHER RECORDED DATA** | |
| Category | Measure |
| Performance status | Australian Modified Karnofsky Performance Status^6^ |
| Frailty | Rockwood Clinical Frailty Scale^7^ |
| Activities of daily living | Barthel Activities of Daily Living^8^ |
| Diagnoses | Diagnoses – incl. all comorbidities according to ICD-10 codes |
| Hospital stays | Admissions, dates, length, fit for discharge status |

Supplementary table 1. Other measures used in this study.

1. Murtagh FE, Ramsenthaler C, Firth A, et al. A brief, patient-and proxy-reported outcome measure in advanced illness: Validity, reliability and responsiveness of the Integrated Palliative care Outcome Scale (IPOS). *Palliative Medicine* 2019;**33**(8):1045-1057.
2. The EuroQol group. EuroQol—a new facility for the measurement of health related quality of life. *Health Policy* 1990;**16**:199–208
3. Morley JE, Malmstrom TK, Miller DK. A simple frailty questionnaire (FRAIL) predicts outcomes in middle aged African Americans. *The journal of nutrition, health & aging* 2012;**16**(7):601-8.
4. Jenkinson C, Coulter A, Bruster S. The Picker Patient Experience Questionnaire: development and validation using data from in-patient surveys in five countries. International *Journal for Quality in Health Care* 2002;**14**(5):353-8.
5. Knapp MRJ, Knudsen HC, Amaddeo F, et al. 2000. Client Socio‐Demographic and Service Receipt Inventory—European Version: development of an instrument for international research. *Br J Psychiatry* 2000;**177**:s28–s33.
6. Abernethy AP, Shelby-James T, Fazekas BS, et al. The Australia-modified Karnofsky Performance Status (AKPS) scale: a revised scale for contemporary palliative care clinical practice. *BMC palliative care* 2005;**4**(1):7
7. Rockwood K, Song X, MacKnight C, et al. A global clinical measure of fitness and frailty in elderly people. *Canadian Medical Association Journal* 2005;**173**(5):489-95
8. Collin C, Wade DT, Davies S, et al. The Barthel ADL Index: a reliability study. *International disability studies*, 1988;***10***(2):61-63.

**Supplementary Information 2.** **Details of qualitative participants and their care preferences.**

Supplementary table 2. Details of qualitative participants.

| **ID** | **Age group** | **CFS**  **t 0, 1, 2** | **Karnofsky**  **t 0, 1, 2** | **Hospital admissions last 6m** | **Hospital admissions in study** | **Lives alone** | **Gender** | **Patient/**  **carer interviewed** | **Interviews** |
| --- | --- | --- | --- | --- | --- | --- | --- | --- | --- |
|  |  |  |  |  |  |  |  |  |  |
| 1 | 80 - 84 | 6/6/6 | 60/60/40 | 3 | 1 | N | F | Y/Y | 2 |
| 2 | 85 - 89 | 6/6/5 | 60/60/60 | 0 | 1 | N | F | Y/N | 3 |
| 3 | 80 - 84 | 5/5/5 | 60/60/60 | 1 | 1 | N | M | Y/N | 3 |
| 4 | 80 - 84 | 5/5/6 | 50/60/50 | 2 | 3 | N | M | Y/Y | 3 |
| 5 | 80 - 84 | 6/6/9 | 40/40/40 | 5 | 1 | N | F | Y/Y | 3 |
| 6 | 80 - 84 | 5/6/6 | 60/60/50 | 1 | 1 | N | M | Y/Y | 1 |
| 7 | 85 - 89 | 6/-/- | 60/-/- | 1 | - | N | F | Y/N | 1 |
| 8 | 90 - 94 | 5/6/5 | 60/60/60 | 1 | 0 | Y | M | Y/N | 3 |
| 9 | 85 - 89 | 6/6/7 | 40/50/50 | 1 | 1 | Y | F | Y/N | 3 |
| 10 | 90 - 94 | 8/7/- | 30/30/0 | 2 | 1 | N | F | N/Y | (3)^1^ |
| 11 | 85 - 89 | 5/5/6 | 50/60/50 | 1 | 0 | N | M | Y/Y | 3 |
| 12 | 80 - 84 | 6/6/5 | 40/60/60 | 1 | 0 | Y | M | Y/Y | 3 |
| 13 | 70 - 74 | 7/6/6 | 50/50/40 | 8 | 7 | Y | F | Y/N | 3 |
| 14 | 90 - 94 | 7/-/7 | 50/-/50 | 2 | 0 | Y | M | Y/N | 2 |
| 15 | 80 - 84 | 6/-/- | 60/0/0 | 3 | 1 | Y | M | Y/N | 1 |
| 16 | 75 - 79 | 7/7/7 | 20/20/40 | 1 | 3 | Y | F | Y/N | 3 |
| 17 | 75 - 79 | 5/5/4 | 50/70/80 | 3 | 3 | N | F | Y/N | 3 |
| 18 | 85 - 89 | 7/-/- | 50/-/- | 1 | - | Y | F | Y/N | 1 |

1. Carer only interviewed

Supplementary table 3: Mixed-methods matrix*.*

| **Preference stability^1^** | **Age**  **(median)** | **AKPS**  **(median)** | **Admissions^2^ (median)** | **Data supporting preference stability** | **Data supporting preference change** |
| --- | --- | --- | --- | --- | --- |
| **Participants with stable preferences**  3 participants  2 female | 85 | 50 | 2 | **Slow recovery with ongoing physical symptoms**  P9 interview 3. ‘Well again the pain. I would think that’s one of the worst things for anybody because it does wear you down in the end’  **Care experiences**  P17 interview 3. ‘I suppose I’ve spent such a lot of my life up the hospitals, you know with my parents… all that sort of thing...’  **Long term aims**  P11 interview 2 ‘Well I want to stay at home as far as possible and uh … we’ve … both signed powers of attorney … so that uh uh if I couldn’t make decisions anymore, then our children could do it for us’ | **Changes in family support**  P9 interview 3 ’well I’m [age 85 – 89] now, so when you get to that age I mean you’re not going to look forward to… it, it would be different if you were living with your family. I think that must be so different… you’ve got your children or perhaps your grandchildren or great grandchildren round you’  **Future events which might affect preferences.**  P17 interview 3. ‘… I’m hoping that it’ll all just go a…that this is all gonna be a big mistake… You know I think… perhaps it will come as a shock if they tell me that yeah you’ve got cancer in two places I don’t know…’ |
| **Participants with semi-stable preferences**  9 participants  5 female | 82 | 50 | 4 | **Care experiences**  P14 interview 3:’Errr how do I prefer… I think the way, the manner in which I’m cared for now is ideal.... From all points you know. From medical point, to social point – with all my family.’  **Concordance with values**  P5 interview 3: ‘O yeah I’ve never liked hospitals. Even when I was well I didn’t like hospitals’ | **Uncertain illness trajectory**  P13 Interview 3. ‘Who knows … I can’t tell; I never know really from day to day what will happen really…’  **Changes in family support**  P12 Interview 2. ‘R: What do you think would be different if you didn’t have that help [from family]? P12: It’d be a hell of a difference, really; I don’t know. And it’s difficult to imagine it, really.’ |
| **Participants with unstable preferences**  2 participants  1 female | 88 | 60 | 1 | **Concordance with values**  P8 Interview 1:  ‘R: Do you think having that fall and going into hospital has changed how you feel about your health or about things?  P8: No I don’t think it has… because I’m an independent sort of chap I think’ | **Life events, family changes, and reluctance to consider the future**  P2 Interview 3. ‘I hope it doesn’t happen but it could be the health of my husband or it could be the health of a family member it it’s … life changes and … goes around and … who knows, who knows what’ll happen. Maybe it’s best not to know… otherwise you’ve got something else to worry about haven’t you’  **Reluctance to consider the future**  P8 interview 2 ‘… I mean I sometimes wonder how I will be in in let’s say 5 years’ time….And then I think, well there’s nothing I can do about it at the moment. I don’t want anybody to commit themselves to whatever I might need in 5 years’ time because we don’t know.’ |

Participants in this table are separated into three groups: those with stable, semi-stable, and unstable preferences. Data supporting preference stability and supporting preference instability is presented for each group to illustrate both supporting and divergent cases.

1. Stable: Most important stable, and importance rating of preference ranked most important is stable (≤1 point change over study)

Semi-stable: Most important stable, but importance rating of preference ranked most important is unstable (≤1 point change over study) **OR** Most important unstable, importance

rating of all preferences ranked as most important is stable.

Unstable: Both most important, and importance rating of preferences ranked most important are unstable

1. Hospitalisations in last year year = 6 months prior to consent and 6 months after consent.

|  | Preference A. extend life | | | Preference B. Improve quality of life | | | Preference C. Remain independent | | | Preference D. Be comfortable | | | Preference E. Support those close to me | | | Preference F. Stay out of hospital | | | Preference G. Other (specify) | | | Ranking – most important  preference | | | Preference stability pattern^2^ |
| --- | --- | --- | --- | --- | --- | --- | --- | --- | --- | --- | --- | --- | --- | --- | --- | --- | --- | --- | --- | --- | --- | --- | --- | --- | --- |
|  | Baseline | 12 week | 24 week | Base  line | 12 week | 24 week | Base  line | 12 week | 24 week | Base  line | 12 week | 24 week | Base  line | 12 week | 24 week | Base  line | 12 week | 24 week | Base  line | 12 week | 24 week | Base  line | 12 week | 24 week |  |
| 1 | 1 | 0 | miss | 4 | 4 | miss | 4 | 4 | miss | 4 | 4 | miss | 4 | 4 | miss | 4 | 4 | miss | n/a | 4 | miss | E | C | miss | Semi stable |
| 2 | 0 | 4 | 0 | 3 | 4 | 0 | 4 | 4 | 4 | 4 | 4 | 4 | 4 | 4 | 4 | miss | 4 | 3 | n/a | 4 | n/a | E | A | E | Unstable |
| 3 | 4 | 4 | 3 | 4 | 4 | 4 | 4 | 4 | 4 | 4 | 4 | 4 | 3 | 4 | 4 | 4 | 4 | 4 | n/a | n/a | n/a | A | F | F | Semi stable |
| 4 | 4 | 4 | 3 | 4 | 4 | 3 | 4 | 4 | 4 | 4 | 4 | 4 | 4 | 4 | 3 | 4 | 4 | 4 | n/a | n/a | n/a | A | A | D | Semi stable |
| 5 | 0 | 2 | 2 | 3 | 4 | 4 | 4 | 4 | 2 | 4 | 4 | 3 | 4 | 4 | 4 | 4 | 4 | 4 | n/a | 4 | n/a | E | F | B | Semi stable |
| 6 | 3 | 3 | 4 | 3 | 2 | 4 | 4 | 3 | 4 | 3 | 3 | 4 | 3 | 3 | 4 | 3 | 4 | 4 | 4 | n/a | n/a | F | F | D | Semi stable |
| 7 | 4 | miss | miss | 4 | miss | miss | 4 | miss | miss | 4 | miss | miss | 4 | miss | miss | 4 | miss | miss | n/a | miss | miss | A | miss | miss | n/a - WITHDREW |
| 8 | 2 | 4 | 2 | 3 | 4 | 3 | 3 | 3 | 2 | 3 | 3 | 3 | 4 | 4 | 2 | 3 | 4 | 2 | 4 | n/a | n/a | E | B | B | Unstable |
| 9 | 1 | 0 | 2 | 4 | 4 | 4 | 4 | 4 | 4 | 2 | 4 | 4 | 2 | 2 | 2 | 4 | 4 | 4 | n/a | n/a | n/a | B | B | B | Stable |
| 10 | n/a | n/a | n/a | n/a | n/a | n/a | n/a | n/a | n/a | n/a | n/a | n/a | n/a | n/a | n/a | n/a | n/a | n/a | n/a | n/a | n/a | n/a | n/a | n/a | n/a PROXY |
| 11 | 4 | 4 | 4 | 4 | 4 | 4 | 4 | 3 | 4 | 4 | 3 | 4 | 4 | 4 | 4 | 4 | 4 | 4 | n/a | n/a | n/a | miss | E | E | Stable |
| 12 | 2 | 3 | 2 | 4 | 1 | 3 | 4 | 4 | 4 | 4 | 3 | 4 | 4 | 3 | 4 | 2 | 3 | 2 | n/a | n/a | n/a | C | E | G^3^ | Semi stable |
| 13 | 3 | 0 | 4 | 2 | 3 | 2 | 4 | 4 | 4 | 3 | 4 | 4 | 3 | 3 | 3 | 2 | 4 | 4 | n/a | n/a | n/a | C | C | G^4^ | Semi stable |
| 14 | 4 | miss | 3 | 4 | miss | 2 | 4 | miss | 3 | 4 | miss | 2 | 4 | miss | 4 | 4 | miss | 3 | n/a | miss | n/a | A | miss | E | Semi stable |
| 15 | 1 | miss | miss | 4 | miss | miss | 4 | miss | miss | 4 | miss | miss | 4 | miss | miss | 4 | miss | miss | n/a | miss | miss | F | miss | miss | n/a RIP |
| 16 | 3 | 3 | 4 | 3 | 4 | 4 | 4 | 4 | 4 | 3 | 3 | 4 | 4 | 4 | 4 | 3 | 1 | 4 | n/a | n/a | n/a | E | E | A | Semi stable |
| 17 | 0 | 0 | 0 | 2 | 2 | 1 | 3 | 4 | 4 | 4 | 2 | 2 | Don’t  know | 4 | 2 | 4 | 4 | 4 | n/a | n/a | n/a | F | F | F | Stable |
| 18 | 0 | miss | miss | 4 | miss | miss | 4 | miss | miss | 4 | miss | miss | 0 | miss | miss | 4 | miss | miss | 4 | miss | miss | G^5^ | miss | miss | n/a LOST |

Supplementary table 4. Qualitative participants’ care preferences.

1. Highlights = changes of ≥2 points.

2. Stable = Most important stable, and importance rating of preference ranked most important is stable (≤1 point change over study)

Semi-stable = Most important stable, but importance rating of preference ranked most important unstable (≤1 point change over study) **OR** Most important unstable, importance rating of all preferences ranked as most important stable.

Unstable = Both most important, and importance rating of preferences ranked most important are unstable

3. Preference G = ‘to maintain mental capacity’. 4. Preference G = ‘to remain financially secure’. 5. Preference G = ‘that I’m in control of my life’

**Supplementary Information 3.** **Distributions of importance ratings of preferences.**

Supplementary figure 2. Histograms showing how the importance ratings of each preference were distributed at each time point. Blue graphs = T0 (baseline) data, Green graphs = T1, and Yellow = T2

**Preference A ‘to extend Life’ Preference B ‘to improve quality of life’**

**Preference C ‘to remain independent’ Preference D ‘to be comfortable’**

**Preference E ‘to support those close to me’ Preference F ‘to stay out of hospital’**

**Supplementary information 4. Missing data report.**

Supplementary table 6. Item non-response for most- important preference

Supplementary table 5. Unit non- response (missing questionnaires)

|  | Time point | | |
| --- | --- | --- | --- |
|  | T0 | T1 | T2 |
| **Unit response** | **82** | **64** | **64** |
| **Unit non-response** | **0** | **18** | **18** |
| Died | 0 | 9 | 12 |
| Withdrew | 0 | 1 | 1 |
| Lost | 0 | 1 | 3 |
| Missed follow up  Due to illness  Declined  Missing – other  Became proxy respondent | 0  0  0  0  0 | 7  3  2  1  1 | 2  0  0  1  1 |

|  | Time point | | |
| --- | --- | --- | --- |
|  | T0 | T1 | T2 |
| **Unit response** | **82** | **64** | **64** |
| Item response | 66 | 53 | 53 |
| Item non response | 16 | 11 | 11 |
| Don’t know | 9 | 5 | 5 |
| Prefer not to say | 3 | 3 | 4 |
| Missing item | 4 | 3 | 2 |

Supplementary table 7. Item non-response for importance rating of each preference

Table 3: Item level missing data for importance of preferred care outcomes

|  | T0 (n = 82) | | | | T1 (n = 64) | | | | T2 (n = 64) | | | |
| --- | --- | --- | --- | --- | --- | --- | --- | --- | --- | --- | --- | --- |
|  | Valid item response | Don’t know | Prefer not to say | Missing | Valid item response | Don’t know | Prefer not to say | Missing | Valid item response | Don’t know | Prefer not to say | Missing |
| Preference A | 71 | 1 | 3 | 7 | 45 | 12 | 3 | 4 | 50 | 6 | 5 | 3 |
| Preference B | 70 | 1 | 3 | 8 | 54 | 4 | 3 | 3 | 56 | 1 | 5 | 2 |
| Preference C | 69 | 2 | 3 | 8 | 57 | 2 | 3 | 2 | 55 | 2 | 5 | 2 |
| Preference D | 72 | 1 | 3 | 6 | 58 | 1 | 3 | 2 | 55 | 1 | 5 | 3 |
| Preference E | 69 | 3 | 3 | 7 | 56 | 2 | 3 | 3 | 55 | 2 | 5 | 2 |
| Preference F | 69 | 2 | 3 | 8 | 57 | 1 | 4 | 2 | 54 | 1 | 5 | 4 |
| Preference G | 14 | 1 | 0 | 67^1^ | 9 | 5 | 0 | 50^1^ | 12 | 0 | 0 | 521 |

1. Includes those who did not specify a preference G, and so didn’t rate its importance
